# Lurasidone‐Associated Urinary Retention in a Patient With Bipolar Affective Disorder: A Case Report

**DOI:** 10.1155/crps/7779671

**Published:** 2026-08-27

**Authors:** Andrew Ridge, Kyle Williams, Bastian Seidel

**Affiliations:** ^1^ University of Tasmania School of Pharmacy and Pharmacology, Hobart, Tasmania, Australia; ^2^ Ochre Health Research Network, Huonville, Tasmania, Australia; ^3^ Older Persons Mental Health Service, Tasmanian Health Service, Hobart, Tasmania, Australia; ^4^ University of Tasmania College of Health and Medicine, Hobart, Tasmania, Australia

**Keywords:** atypical antipsychotics, bipolar affective disorder, lurasidone, urinary retention

## Abstract

Atypical antipsychotics, including lurasidone, are increasingly prescribed due to their perceived lower risk of side effects. We present the case of a 65‐year‐old woman with longstanding BPAD who developed symptoms of urinary retention (UR), confirmed on bladder ultrasound, soon after beginning lurasidone monotherapy for bipolar affective disorder (BPAD) management. Despite dose reduction and addition of tamsulosin, symptoms of UR persisted and only resolved with cessation of lurasidone. The causal relationship between lurasidone and UR was assessed using the Naranjo Adverse Drug Reaction (ADR) Probability Scale, yielding a score of 8, indicating a highly ‘probable’ ADR. A literature review revealed only two prior reports of lurasidone‐associated UR, highlighting the rarity of this presentation. This case underscores the need for clinicians to remain vigilant for UR in patients prescribed lurasidone, or other atypical antipsychotics, despite their generally favourable side effect profile. Early recognition and management are critical to prevent complications and ensure appropriate ongoing treatment of BPAD.

## 1. Introduction

Bipolar affective disorder (BPAD) is a recurrent illness characterised by episodes of depression and mania [1] and is associated with higher morbidity and mortality for the individual [2]. Prompt diagnosis and effective treatment of BPAD may improve disease trajectory, reduce morbidity and associated healthcare utilisation costs [3, 4].

Lithium is often considered the first‐line agent for acute BPAD symptoms and prophylactic therapy but is also associated with an array of well‐known adverse effects. More recently, both first‐generation and atypical antipsychotics have been used in the management of BPAD [5]. Atypical agents, including lurasidone, are viewed as a safer alternative because of the lower incidence of extrapyramidal side effects [6, 7]. Unlike other atypical agents, lurasidone has low affinity for H_1_, M_1_ and 5‐HT_2C_ receptors which are associated with sedation, increased weight and metabolic syndrome [8, 9]. In Australia and internationally, lurasidone is recommended as monotherapy and an adjunctive treatment for acute bipolar depression [10–12].

Many drugs may cause urinary retention (UR), but there are a few reports of this being associated with lurasidone. We report a case of UR associated with the atypical antipsychotic lurasidone.

This case presentation adheres to the CARE standards of reporting. The University of Tasmania Human Research Ethics Committee (HREC) endorses this case report and considers it a quality initiative. As such, a formal HREC review was waived in accordance with applicable ethical guidelines. Case Report ID: UTASCR001

## 2. Case Presentation

Presented here is the case of a 65‐year‐old woman (LF) with a history of BPAD. She was initially diagnosed with BPAD approximately 37 years ago, after the birth of a child in the 1980s. She was prescribed immediate‐release lithium carbonate tablets (lithium), eventually stabilising on 875 mg/day by 2002. A good clinical response and two decades of stable mental health without adverse effects followed. Her other medical history was unremarkable (but included osteoarthritis and cataracts), and her only other medication (a combined oral contraceptive) had been ceased since at least 2012. She moved to a rural/remote area of Tasmania, Australia, in 2012 with her supportive husband, continued to work in an administrative role, and maintained regular contact with her general practitioner (GP).

In 2021, LF developed symptoms often associated with lithium toxicity (including glossitis, xerostomia, hypercalcaemia, hyperparathyroidism and chronic kidney disease [CKD]). LF requested that she be weaned off lithium, and under the supervision of her GP and psychiatrist, this was achieved in 2022. She felt ‘much better’, and she maintained a ‘stable’ mental state for several years. She suffered a femoral condyle fracture and a displaced radial fracture, thought to be caused by lithium‐induced hyperparathyroidism. Based on past negative experiences with medication (viz., lithium), LF declined treatment of her hyperparathyroidism, and a partial parathyroidectomy was performed in mid‐2024. She made a seemingly normal recovery immediately post‐surgery.

Two weeks after the parathyroidectomy, she presented to her new GP with low mood, nihilistic thoughts, anhedonia, poor oral intake and weight loss (8–10 kg), psychomotor retardation and anxiety. After attending two different emergency departments over the next weeks, no abnormal pathology results were found to explain her condition, and her symptoms steadily worsened.

Four weeks after the parathyroidectomy, LF was urgently admitted to the mental health inpatient unit of a local hospital. Lurasidone (40 mg at night) and lorazepam (0.5–1.0 mg every 4 h, if needed) were commenced over the following days with good effect; there was decreased anhedonia and improved mood, energy levels, motivation and concentration. She was transferred to a low‐acuity mental health unit before being discharged 15 days later taking lurasidone (60 mg at night), lorazepam (0.5 mg twice a day) and quetiapine (25 mg every 4 h, if needed), although she did not actually take the latter two drugs. Adherence to the prescribed medication regimen was considered likely based on supervision from her husband, use of one prescriber and pharmacy (after hospital discharge), and the level of insight retained by LF. Absence of use of PRN quetiapine was reported by LF, with no evidence to suggest inaccuracy in her reporting.

Four weeks after discharge, LF presented to a GP experiencing a ‘weak urinary stream’, difficulty emptying her bladder and amotivation. UR was suspected, and a bladder ultrasound confirmed pre‐ and post‐void bladder volumes of 554 and 456 mL, respectively. The dose of lurasidone was reduced to 40 mg and tamsulosin (400 µg daily) was added, with subsequent symptomatic relief (i.e., less abdominal discomfort), but she experienced ongoing urinary hesitancy and retention.

The community mental health service was contacted for advice, and 4 days later during a home‐visit, a nurse found the patient’s mental state had deteriorated to the point where she was unable to talk, lacked motivation, wandered aimlessly, had poor self‐care and was at times almost catatonic. There was one episode of significant urinary incontinence. Despite reluctance to again travel 3 h to the hospital, the patient was eventually admitted with a depressive episode of BPAD and ongoing UR. She had self‐ceased lurasidone upon presentation to the hospital. Her depressed mood continued, and over the next weeks, LF had nine sessions of ECT before she clinically improved and was discharged home. She was discharged with lorazepam and tapering of her acute course of electroconvulsive therapy as an outpatient.

Options for BPAD prophylaxis were discussed with LF (viz., lithium, a different atypical antipsychotic, or another mood stabiliser). Low‐dose lithium (65.5 mg at night) was accepted by LF, with the recommendation that she have regular renal function and lithium level monitoring. Lorazepam was reduced to 0.25 mg at night and then ceased. Over the following months, the lithium dose was increased to 125 mg daily (serum levels remained <0.2 mmol/L), and olanzapine 2.5 mg daily was added following possible early signs of a depressive episode emerging. Her eGFR remained relatively stable at approximately 40 mL/min; however, therapeutic lithium levels could not be achieved in the context of CDK. With this, and worsening depression, lithium was ceased and LF accepted the recommendation to complete a further acute course of ECT to remit her depressive episode, with the view for maintenance ECT to follow as BPAD prophylaxis.

She experienced ‘frothy’ urine, constipation and perceived changes in urinary patterns while taking olanzapine, although these resolved when LF stopped taking olanzapine. Ongoing maintenance ECT was well tolerated and effective, and LF has remained euthymic, cognitively intact, gaining weight and enjoying many activities she previously enjoyed.

## 3. Discussion

The case described here is a female patient with BPAD who developed UR in association with lurasidone monotherapy and is noteworthy as this drug is understood to cause little or no anticholinergic side effects [13].

While UR in the setting of severe psychomotor impairment or catatonia have been the subject of case reports [14–16], no micturition changes were noted by the GP at the first presentation or during her initial hospitalisation period. The onset of urinary symptoms correlates well with the initiation of lurasidone, and this was considered the most likely cause of UR in this case.

We used the Naranjo Algorithm Adverse Drug Reaction (ADR) Probability Scale [17] to assess the causal relationship between lurasidone and the observed UR. A Naranjo score of 8 here suggests ‘probable’ causation of the ADR by lurasidone. The scoring algorithm for the Naranjo scale is shown in Table 1.

**Table 1:** Naranjo scale for assessing adverse drug reaction probability (adapted from Naranjo et al. [17]).

Naranjo scale item	Yes	No	Do not know or not done
1. Are there previous conclusive reports on this reaction?	+1	—	—
2. Did the adverse events appear after the suspected drug was given?	+2	—	—
3. Did the adverse reaction improve when the drug was discontinued or a specific antagonist was given?	+1	—	—
4. Did the adverse reaction appear when the drug was re administered?	—	—	0
5. Are there alternative causes that could have caused the reaction?	—	+2	—
6. Did the reaction reappear when a placebo was given?	—	—	0
7. Was the drug detected in any body fluid in toxic concentrations?	—	0	—
8. Was the reaction more severe when the dose was increased, or less severe when the dose was decreased?	+1	—	—
9. Did the patient have a similar reaction to the same or similar drugs in any previous exposure?	—	0	—
10. Was the adverse event confirmed by any objective evidence?	+1	—	—
Total	8		

*Note*: Scoring for Naranjo scale: ≥9 = definite ADR; 5–8 = probable ADR; 1–4 = possible ADR; 0 = doubtful ADR.

Lurasidone was approved for the treatment of schizophrenia in 2014 in Australia [18]. Like other atypical antipsychotic agents, lurasidone exhibits D_2_ receptor antagonism, but also antagonises serotoninergic receptor activity at 5HT_7_ and 5HT_2A_ receptors and is a partial agonist of 5HT_1A_ [9, 19], leading to enhancement of noradrenergic and dopaminergic neurotransmission [5]. It is believed to have negligible muscarinic and histaminic activity and thus have fewer adverse effects on the autonomic nervous system [9, 13]. A weak to moderate affinity for α_1_ adrenergic receptors has also been reported [9, 20]. The variety of receptor sites targeted by atypical antipsychotic agents should result in a more favourable side effect profile when compared to first‐generation antipsychotics, especially with respect to the occurrence of extrapyramidal and anticholinergic side effects.

A literature search found two reports of lurasidone‐associated UR; one involving lurasidone monotherapy [21] and one in combination with other psychoactive agents [22]. A systematic review by Faure Walker et al. did not list lurasidone as being associated with UR, although there was an association with other atypical agents (e.g., olanzapine and quetiapine) [23].

As in the scenario presented by Varghese et al. [21], the case here is of a female with no history of UR, and symptoms improved with dose reduction. The authors agree with the proposed mechanism of lurasidone‐induced UR; like other atypical antipsychotic agents, lurasidone exhibits D_2_ receptor antagonism but also antagonises serotoninergic receptor activity at 5HT_7_ and activates α1 receptors [19], potentially leading to UR. Unlike lurasidone, olanzapine is known to have a strong affinity for D_1_, D_2_, D_4_, H_1_ and M_1_ receptors, which may explain why LF developed changed micturition patterns when using olanzapine [24]. There are multiple receptor types and pathways involved in micturition and bladder control, and the subtle differences between lurasidone’s and olanzapine’s actions are a reflection of this.

A challenge for clinicians treating patients with BPAD who develop UR is—what to use instead of lurasidone? Other atypical antipsychotics are not without risk [23], and other common treatments have their own palette of adverse effects. Persisting with a lower dose of lurasidone in our patient did not fully alleviate her symptoms, nor did adding a α‐1A antagonist, as suggested by Asnis [25]. Ultimately, the gold‐standard for severe, treatment‐resistant bipolar depression— ECT [26]—was used with a positive clinical outcome for our patient.

## 4. Conclusion

Given that this is only the second case of UR associated with lurasidone monotherapy, prescribers should remain vigilant for signs and symptoms suggestive of UR in patients prescribed this agent. Similar caution should also be used among patients using other atypical antipsychotic agents despite the perception that they have less risk of anticholinergic effects.

## Author Contributions

**Andrew Ridge**: data collation, draft manuscript, review and editing, documentation. **Kyle Williams**: clinical care, draft manuscript, review and editing. **Bastian Seidel**: clinical care, conceptualisation, review and editing.

## Funding

No funding was received for this manuscript. Open access publishing facilitated by University of Tasmania, as part of the Wiley ‐ University of Tasmania agreement via the Council of Australasian University Librarians.

## Disclosure

The manuscript has been prepared with reference to and meets the CARE Case Reporting Guidelines. The guidelines are available via publisher upon request. All authors have read and approved the final manuscript.

## Ethics Statement

The University of Tasmania Human Research Ethics Committee (HREC) endorses the case report and considers it a quality initiative. As such, a formal HREC review is waived in accordance with applicable ethical guidelines. Case Report ID: UTASCR001.

## Consent

Written informed consent was obtained from the patient for the publication of this case report. A copy of the written consent is available for review by the Editor‐in‐Chief of this journal.

## Conflicts of Interest

The authors declare no conflicts of interest.

## Data Availability

All data generated or analysed during this study are included in this published article.
